# Functional genomic approaches to elucidate the role of enhancers during development

**DOI:** 10.1002/wsbm.1467

**Published:** 2019-12-05

**Authors:** Genevieve E. Ryan, Emma K. Farley

**Affiliations:** ^1^ Department of Medicine University of California San Diego California; ^2^ Division of Biological Sciences, Department of Medicine University of California San Diego California

**Keywords:** development, enhancers, genomics

## Abstract

Successful development depends on the precise tissue‐specific regulation of genes by enhancers, genetic elements that act as switches to control when and where genes are expressed. Because enhancers are critical for development, and the majority of disease‐associated mutations reside within enhancers, it is essential to understand which sequences within enhancers are important for function. Advances in sequencing technology have enabled the rapid generation of genomic data that predict putative active enhancers, but functionally validating these sequences at scale remains a fundamental challenge. Herein, we discuss the power of genome‐wide strategies used to identify candidate enhancers, and also highlight limitations and misconceptions that have arisen from these data. We discuss the use of massively parallel reporter assays to test enhancers for function at scale. We also review recent advances in our ability to study gene regulation during development, including CRISPR‐based tools to manipulate genomes and single‐cell transcriptomics to finely map gene expression. Finally, we look ahead to a synthesis of complementary genomic approaches that will advance our understanding of enhancer function during development.

This article is categorized under:

Physiology > Mammalian Physiology in Health and Disease
Developmental Biology > Developmental Processes in Health and Disease
Laboratory Methods and Technologies > Genetic/Genomic Methods

## Graphical abstract

The correct spatiotemporal regulation of genes by enhancers is critical for successful development from a single fertilized egg to a complex multicellular organism. Herein, we discuss current genome‐wide approaches to identify enhancers and to dissect enhancer function. Figure created with BioRender.

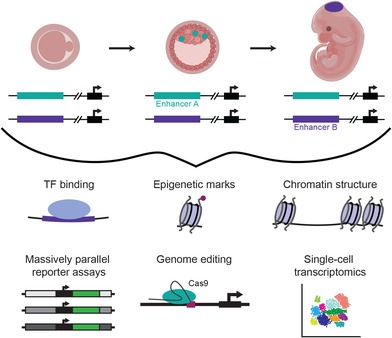

## 1 INTRODUCTION

Animal development is the dynamic process through which a single fertilized egg ultimately gives rise to a multitude of distinctive cell types. Cells maintain unique identities and are able to carry out specialized functions by expressing different sets of genes. Enhancers are *cis**‐***regulatory elements that direct precise, tissue‐ and cell type‐specific patterns of gene expression. The correct spatiotemporal regulation of genes by enhancers is critical for successful development (Levine, 2010). Enhancers were first recognized over 30 years ago as being short DNA sequences that positively regulate expression of a gene regardless of their position or orientation relative to the transcription start site (Banerji, Rusconi, & Schaffner, 1981; Moreau et al., 1981; Shlyueva, Stampfel, & Stark, 2014). Although decades of research have focused on identifying and validating enhancers, we still do not know how many enhancers exist in any genome and currently lack a broad understanding of which sequences within an enhancer are important for function. Enhancers contain hundreds of nucleotides and several transcription factor (TF) binding sites, so deciphering how enhancer sequences encode function is a complex problem. Because enhancers can act over distances on the order of a megabase to regulate target gene expression (Amano et al., 2009), identifying which gene or genes a given enhancer regulates can be far from straightforward. Furthermore, enhancer function can be conserved without discernible sequence conservation (Blow et al., 2010; Meireles‐Filho & Stark, 2009), making it difficult to distill general principles of enhancer activity using comparative genomics in spite of significant efforts.

Despite these challenges in studying enhancer function, substantial progress has been made in recent years due to advances in sequencing technology and high‐throughput methodologies to identify and validate candidate enhancers. On the order of one million candidate enhancers have been identified in the human genome, based on genome‐wide interrogation of TF binding sites and chromatin signatures associated with active enhancers (Dunham et al., 2012; Humbert et al., 2012). Furthermore, there is an increasing appreciation that common variants associated with disease disproportionately reside within putative enhancer sequences (Corradin & Scacheri, 2014; Ernst et al., 2011; Wu & Pan, 2018). Thus, understanding how enhancers function has become an area of significant interest, not only for their roles in normal development, but also because linking enhancer sequence directly to function could provide unprecedented insight into the genetic basis of disease and evolutionary adaptation. With significant advances in sequencing and genome editing technologies, we have never been in a better position to identify and functionally validate candidate enhancers, but there remain significant bottlenecks in our ability to comprehensively understand enhancer function. In this review, we discuss current methodologies used to identify putative enhancers and the techniques that have been developed to study their function at scale. We also review two recent waves of literature that (a) use genome editing tools to interrogate candidate enhancer sequences and (b) utilize single‐cell RNA sequencing to construct developmental atlases of whole embryos. Finally, we look ahead toward a synthesis of these complementary approaches, which will broadly expand our knowledge of how enhancer sequences encode precise control of gene expression during development.

## 2 GENOME‐WIDE METHODOLOGIES TO IDENTIFY PUTATIVE ENHANCERS

Over the last decade, next‐generation sequencing technologies have revolutionized our ability to identify putative enhancers throughout whole genomes. Pairing classic molecular genetic approaches with high‐throughput sequencing has enabled the rapid construction of genome‐wide maps of TF binding sites, histone modifications, nucleosome positions, and long‐range genomic interactions. These efforts were spearheaded by the Encyclopedia of DNA Elements (ENCODE) Consortium (Dunham et al., 2012), and led to the identification of chromatin signatures that are strongly associated with active enhancers. Furthermore, these data demonstrated that single nucleotide polymorphisms associated with disease reside disproportionately within candidate enhancers (Corradin & Scacheri, 2014; Ernst et al., 2011; Wu & Pan, 2018), highlighting the importance of elucidating how enhancer sequences contribute to development, homeostasis, and disease.

### 2.1 Genome‐wide identification of TF binding sites by ChIP‐seq

**Figure 1:**
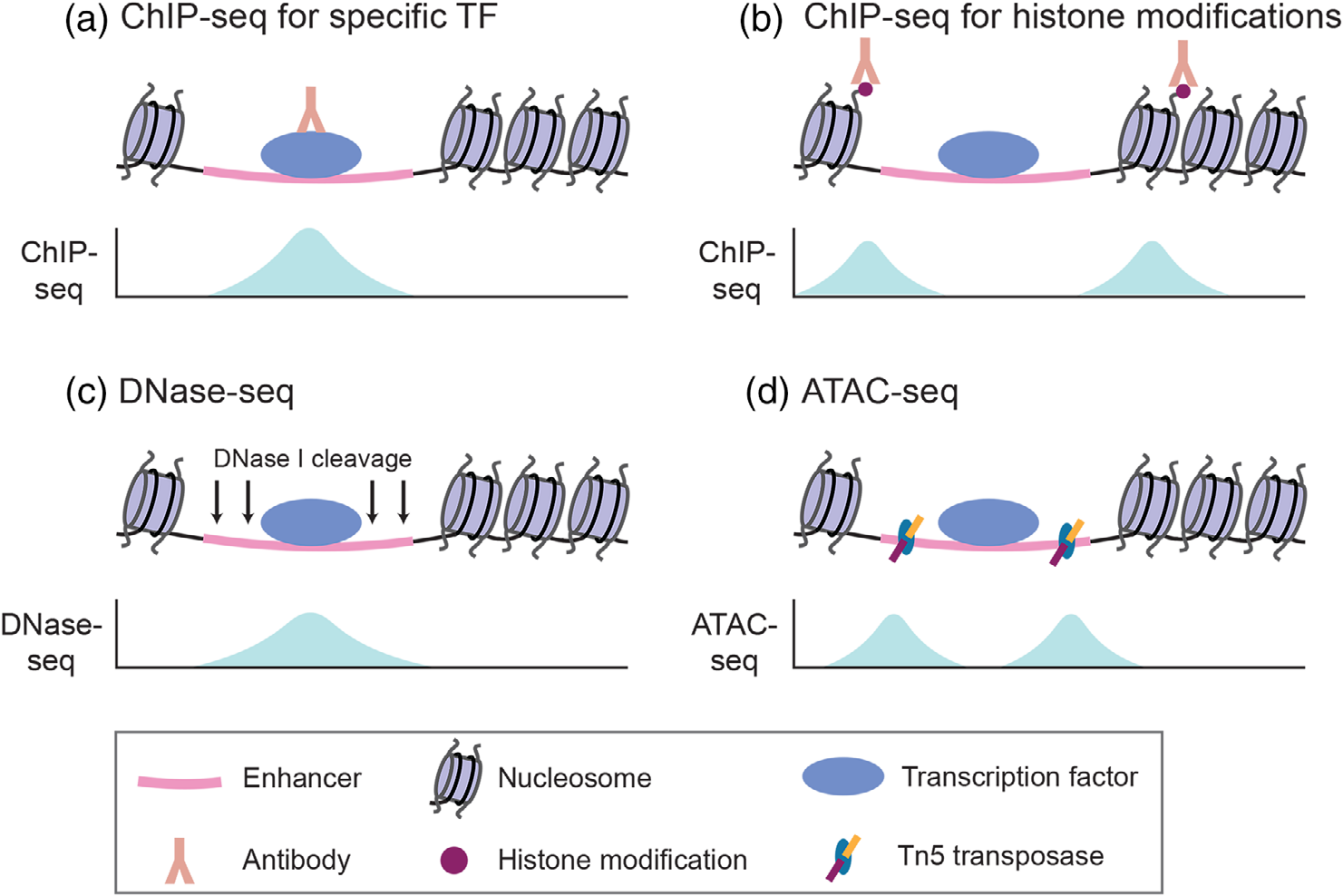
Genome‐wide methods to identify putative enhancers. The basic principle of each experimental approach and the corresponding data readout is shown for each method. (a) Chromatin immunoprecipitation followed by high‐throughput sequencing (ChIP‐seq) uses antibodies targeting a specific TF to determine the location of its binding sites genome‐wide. The broad peaks generated by ChIP‐seq cannot determine precise footprints of TF binding, but this can be achieved by adding a 5′ to 3′ exonuclease digestion step to the protocol (ChIP‐exo). (b) Nucleosomes flanking active enhancers often carry stereotypical histone modifications (e.g., H3K4me1 and H3K27ac) that can be detected with specific antibodies by ChIP‐seq. (c) Active enhancers and other *cis*‐regulatory elements are found within open chromatin that is depleted of nucleosomes. Accessible chromatin can be detected by DNase I digestion followed by high‐throughput sequencing (DNase‐seq). (d) Accessible chromatin can also be detected by assay for transposase‐accessible chromatin using sequencing (ATAC‐seq), where Tn5 transposase simultaneously fragments and tags accessible DNA prior to sequencing

Chromatin immunoprecipitation followed by deep sequencing (ChIP‐seq; Johnson, Mortazavi, Myers, & Wold, 2007; Robertson et al., 2007) has been used extensively to identify genome‐wide binding of TFs, both in vitro and in vivo (Figure 1a). In ChIP‐seq, chromatin is crosslinked using formaldehyde, which covalently links TFs to their native binding sites. The chromatin is then sheared and immunoprecipitated using antibodies specific to a TF of interest. Following immunoprecipitation, the crosslink is reversed and DNA is purified, sequenced, and computationally analyzed to identify TF binding sites. For a given TF, ChIP‐seq will typically identify thousands of regions bound by TFs, which are predominantly found in promoters, introns, and intergenic regions (X. Y. Li et al., 2008; Medina et al., 2008). ChIP‐seq is also able to identify changes in TF occupancy of binding sites throughout development (Gentleman et al., 2010; Hagman et al., 2010; Pilon et al., 2011), reflecting that TF binding is dynamic and highly specific to developmental stage.

ChIP‐seq has been extremely useful for identifying the genomic regions bound by a given TF, including putative enhancer sequences. However, several studies have demonstrated that TF binding identified by ChIP‐seq does not necessarily correspond to active *cis*‐regulatory elements (Kvon, Stampfel, Omar Yáññez‐Cuna, Dickson, & Stark, 2012; X. Y. Li et al., 2008; MacArthur et al., 2012), suggesting that TFs can bind DNA without having a functional role in gene expression. There are several possible explanations for this observation. First, because TFs typically bind regulatory elements in a combinatorial manner (Halfon et al., 2000; Sandmann et al., 2007; Yuh, Ransick, Martinez, Britten, & Davidson, 1994), the binding of any one TF may not be sufficient to activate transcription. Because TFs often bind in complexes, a TF of interest may be immunoprecipitated in ChIP without being directly bound to DNA. TFs also have a general affinity for DNA (Hammar et al., 2012), and may bind transiently to accessible DNA outside of functional contexts and regardless of whether a specific binding motif is present. ChIP‐seq relies on formaldehyde crosslinking and thus transient DNA–protein interactions could be captured by this technique. Indeed, even proteins that do not bind DNA are enriched at highly transcribed genes by ChIP‐seq (Teytelman, Thurtle, Rine, & van Oudenaarden, 2013). Recently, alternatives to ChIP‐seq that eliminate the need for crosslinking have been developed, including Cleavage Under Targets and Release Using Nuclease (CUT&RUN; Skene & Henikoff, 2017) and Cleavage Under Targets and TAGmentation (CUT&TAG; Kaya‐okur et al., 2019). Like ChIP‐seq, these approaches both employ a TF‐specific antibody to identify protein–DNA interactions. However, they also utilize a DNA‐cutting enzyme fused to Protein A, which binds specifically to Immunoglobin G and thus targets antibody‐bound TFs. In CUT&RUN, a Protein A‐MNase fusion protein cleaves DNA on either side of a TF, releasing TF‐DNA complexes into solution for downstream purification and library preparation steps. CUT&TAG improves upon this methodology by employing a Protein A‐Tn5 transposase fusion protein loaded with sequencing adaptors, generating DNA fragments that can be directly amplified and sequenced. Both methods are performed in situ, allow for low starting cell numbers, and produce substantially decreased background compared to ChIP‐seq.

In ChIP‐seq datasets, false positives can also arise due to experimental factors including chromatin fragment size (Rye, Sætrom, & Drabløs, 2011), read depth (Jung et al., 2014), and signal cutoff thresholds (Gomes et al., 2014). Choice of signal cutoff thresholds can be particularly important when analyzing ChIP‐seq data. If set too low, the false‐positive rate will increase but if set too high, it may not be possible to capture functional interactions that are highly dynamic or low‐affinity (Gomes et al., 2014; Landt et al., 2012). Although ChIP‐seq datasets contain both high‐ and low‐affinity TF binding sites, there is a tendency to focus on high‐affinity sites and to ignore or exclude lower affinity sequences during analysis (Nettling, Treutler, Cerquides, & Grosse, 2016). Models of TF binding specificity, such as position–weight matrices, are commonly used to predict functional TF binding sites within regions identified by ChIP‐seq. Sequences with higher affinity scores are presumed to have a greater likelihood of being functional binding sites, and many algorithms use seemingly arbitrary default cutoff thresholds, for example, the 0.8 relative log‐likelihood threshold recommended by JASPAR, an open‐source database of TF binding site profiles (Sandelin, 2004). While it is important to prioritize ChIP‐seq peaks, focusing on high affinity sites may be shortsighted, particularly because developmental enhancers are highly dynamic and often contain low‐affinity or otherwise suboptimal binding sites (Crocker et al., 2015; Farley et al., 2015; Farley, Olson, & Levine, 2016; Hosokawa et al., 2018). Many studies have recently demonstrated that suboptimal TF binding sites within enhancers can be a requirement for precise gene expression and biological function during development (Crocker et al., 2015; Crocker, Preger‐Ben Noon, & Stern, 2016; Farley et al., 2015; Farley, Levine, Olson, Zhang, & Rokhsar, 2016; Ramos & Barolo, 2013; Rowan et al., 2010). Because TFs bind degenerate DNA sequences, it remains a significant challenge to discriminate functional TF binding sites from background sequences in ChIP‐seq data. This issue can be overcome by generating position–weight matrices from datasets depleted of high‐affinity binding sites, which is more accurate at predicting low‐affinity binding sites, and surprisingly even high‐affinity binding sites, compared to conventional methods (Zandvakili, Campbell, Gutzwiller, Weirauch, & Gebelein, 2018). While ChIP strategies have identified novel enhancers (Heintzman et al., 2007), it will be important to improve our ability to identify low‐affinity TF binding and to differentiate between functional and non‐functional binding, particularly when interrogating the genome for developmental enhancers.

The utility of ChIP‐seq for the study of enhancer sequences is also limited by its resolution. In standard ChIP‐seq protocols, chromatin is randomly sheared by sonication to produce fragments within the range of 200–500 basepairs (Mahony & Pugh, 2015). The resolution of ChIP‐seq is similar to fragment size, on the order of hundreds of basepairs, while TF binding motifs are typically 6–20 basepairs (Afek, Schipper, Horton, Gordân, & Lukatsky, 2014). Thus, ChIP‐seq is unable to resolve individual TF binding events within a putative enhancer sequence. Furthermore, a TF binding motif can be found many times within a region identified by ChIP‐seq, and in these instances it is not known which motifs may be functional. ChIP‐exo is a method that provides improved resolution by adding a 5′ to 3′ exonuclease digestion step to conventional ChIP‐seq protocols, allowing the region bound by the TF to be isolated (Rhee & Pugh, 2011). Exonuclease activity is impeded by crosslinked proteins, creating a 5′ border on either side of bound TFs that can be used to precisely map TF binding sites (Rhee & Pugh, 2011). In contrast to ChIP‐seq, ChIP‐exo enables single nucleotide resolution of TF binding and reveals exact footprints of occupied TF binding sites. Because the exonuclease used in ChIP‐exo digests unbound DNA that can contaminate immunoprecipitates, this method also has an improved signal to noise ratio compared to ChIP‐seq (Rhee & Pugh, 2011). Despite these advantages of ChIP‐exo, this method is not yet widely used and thus most genome‐wide TF binding data has been obtained by ChIP‐seq, which captures relatively broad windows of TF binding. While ChIP‐seq methods to identify TF binding have certainly advanced our ability to locate putative *cis*‐regulatory elements in the genome, standard protocols lack the resolution to identify precise binding sites within an enhancer. Furthermore, it still remains unclear which binding events regulate transcription and are functional.

### 2.2 ChIP‐seq to identify transcriptional cofactor localization genome‐wide

ChIP‐seq does not rely on direct protein–DNA interactions, and thus can be used to identify cofactor proteins associated with active enhancers. Cofactors typically do not bind DNA directly, but are recruited to *cis*‐regulatory elements by TFs, where they can act to repress or activate transcription. The transcriptional coactivator protein p300 has been particularly useful for identifying active enhancers. p300 acts as an adaptor for protein–protein interactions and also has histone acetyltransferase activity (Visel et al., 2009), which relaxes the chromatin making it accessible to TFs and the basal transcriptional machinery. Several studies in mice and humans have demonstrated that p300 binding can be used to predict >80% of tissue‐specific enhancer activity (Blow et al., 2010; May et al., 2012; Visel et al., 2009, 2013). While p300 provides an excellent indicator of active enhancers, binding of a coactivator is not sufficient to understand why a particular sequence activates transcription, or which mutations may impact enhancer function. To further improve identification of active enhancers, the colocalization of p300 with other chromatin features, including histone modifications, is often used to locate putative enhancers.

### 2.3 ChIP‐seq for histone modifications associated with active enhancers

Genome‐wide mapping of histone modifications using ChIP‐seq has revealed patterns that are stereotypically found at active enhancers (Figure 1b; Bonn et al., 2012; Heintzman et al., 2007, 2009; Rada‐Iglesias et al., 2011; Roh, Cuddapah, & Zhao, 2005). While enhancers are often associated with H3K4me1 and promoters are often associated with H3K4me3, both enhancers and promoters tend to be marked by H3K27ac when active and H3K27me3 when repressed (Arnold et al., 2013; Bonn et al., 2012; Peters et al., 2002; Rada‐Iglesias et al., 2011). Together, H3K4me1 and H3K27ac are widely used to predict active enhancers (Bonn et al., 2012; Kharchenko et al., 2011; Rada‐Iglesias et al., 2011; Shen et al., 2012; Wamstad et al., 2012). Interestingly, some enhancers are associated with bivalent chromatin (Bernstein et al., 2006; Vastenhouw et al., 2010), that is, the presence of both H3K4me1 and H3K27me3. This combination of activating and repressive marks is characteristic of poised enhancers (Rada‐Iglesias et al., 2011), which are common in development (Creyghton et al., 2010). H3K79me3, which is most commonly found within actively transcribed gene bodies, has also been found at active enhancers (Barski et al., 2007; Bonn et al., 2012). In addition to epigenetically modified histones, the histone variants H2A.Z and H3.3 have been associated with putative enhancers, particularly when found together within a histone octamer (C. Jin et al., 2009).

Despite the associations between these histone modifications and enhancers, there is currently no consensus regarding which combination of histone modifications should be used to identify active enhancers. Furthermore, the histone modifications found at a given enhancer may be context‐specific. For example, latent enhancers are characterized by the absence, and then acquisition, of H3K4me1 and H3K27ac following stimulation of cell signaling pathways (Ostuni et al., 2013). None of the known histone modifications associated with enhancers correlate perfectly with enhancer activity, and even combinations of histone marks are unable to accurately predict all active enhancers (Arnold et al., 2013; Bonn et al., 2012). Indeed, many active enhancers lack characteristic histone modifications. For example, >40% of mesodermal enhancers in *Drosophila melanogaster* embryos could not be predicted based on the presence of H3K27ac (Bonn et al., 2012). It also remains unclear whether histone modifications have a direct role in regulating transcription, or whether they are simply useful indicators of where enhancers may reside within the genome.

### 2.4 Genome‐wide assays for chromatin accessibility

Active enhancers are depleted of nucleosomes, and thus assays for nucleosome positioning and chromatin accessibility are also widely used to identify putative enhancer sequences. Classic molecular biological techniques in which accessible DNA is cleaved by DNase I or micrococcal nuclease (MNase) have been paired with next‐generation sequencing to generate genome‐wide maps of chromatin accessibility. DNase‐seq is based on DNase footprinting, in which DNase I hypersensitive sites are digested and DNA bound by TFs and other proteins is protected (Figure 1c). While DNase footprinting traditionally analyzes digestion products by Southern Blot, in DNase‐seq a linker sequence is added to the protected DNA following DNase digestion to enable subsequent sequencing (Boyle et al., 2008; Humbert et al., 2012). This method generates basepair resolution of DNase I digestion, and thus footprints of TF binding. MNase‐seq utilizes micrococcal nuclease digestion, which cuts the linker DNA between adjacent nucleosomes (Schones et al., 2008; Valouev et al., 2011). Nucleosomal DNA is protected from digestion, and is subsequently purified and sequenced to reveal nucleosome positions genome‐wide. More recently, accessible chromatin has been studied by assay for transposase‐accessible chromatin using sequencing (ATAC‐seq), in which sequencing adaptors are directly transposed into native chromatin (Figure 1d; Buenrostro, Giresi, Zaba, Chang, & Greenleaf, 2013). ATAC‐seq is carried out as a simple two‐step protocol that involves insertion of a hyperactive Tn5 transposase (Adey et al., 2010; Goryshin & Reznikoff, 1998) carrying sequencing adaptors, which simultaneously fragments and tags DNA, followed by polymerase chain reaction (PCR). This method gives basepair resolution of nucleosome‐depleted genomic regions, and can be carried out rapidly because it does not depend on separate enzymatic digestion and adaptor ligation steps (Buenrostro et al., 2013).

Chromatin accessibility assays are advantageous because they can predict *cis*‐regulatory regions independent of any specific TF, cofactor, or histone modification. However, these methods identify promoters and insulators as well as enhancers. Furthermore, open chromatin does not necessarily mean that a given DNA sequence is active or functional. Enhancers located in open chromatin can be held in an inactive state by repressive TFs, which are common in development (Gray & Levine, 1996). For example, enhancers with cell type‐specific regulatory activity have been found to be open and accessible in diverse cell types in which the enhancer is not active (Arnold et al., 2013). Repressed enhancers found within accessible chromatin may also be primed for activity at a later developmental timepoint (Zaret & Carroll, 2011). While genome‐wide profiling of chromatin accessibility cannot detect specific *cis‐*regulatory functions, these techniques are particularly powerful when combined with ChIP‐seq data. The overlay of transcriptional coactivators, histone modifications associated with enhancers, and chromatin accessibility at a given genomic locus can provide strong correlative evidence of active enhancers.

### 2.5 Genome‐wide assays for chromosomal interactions

**Figure 2:**
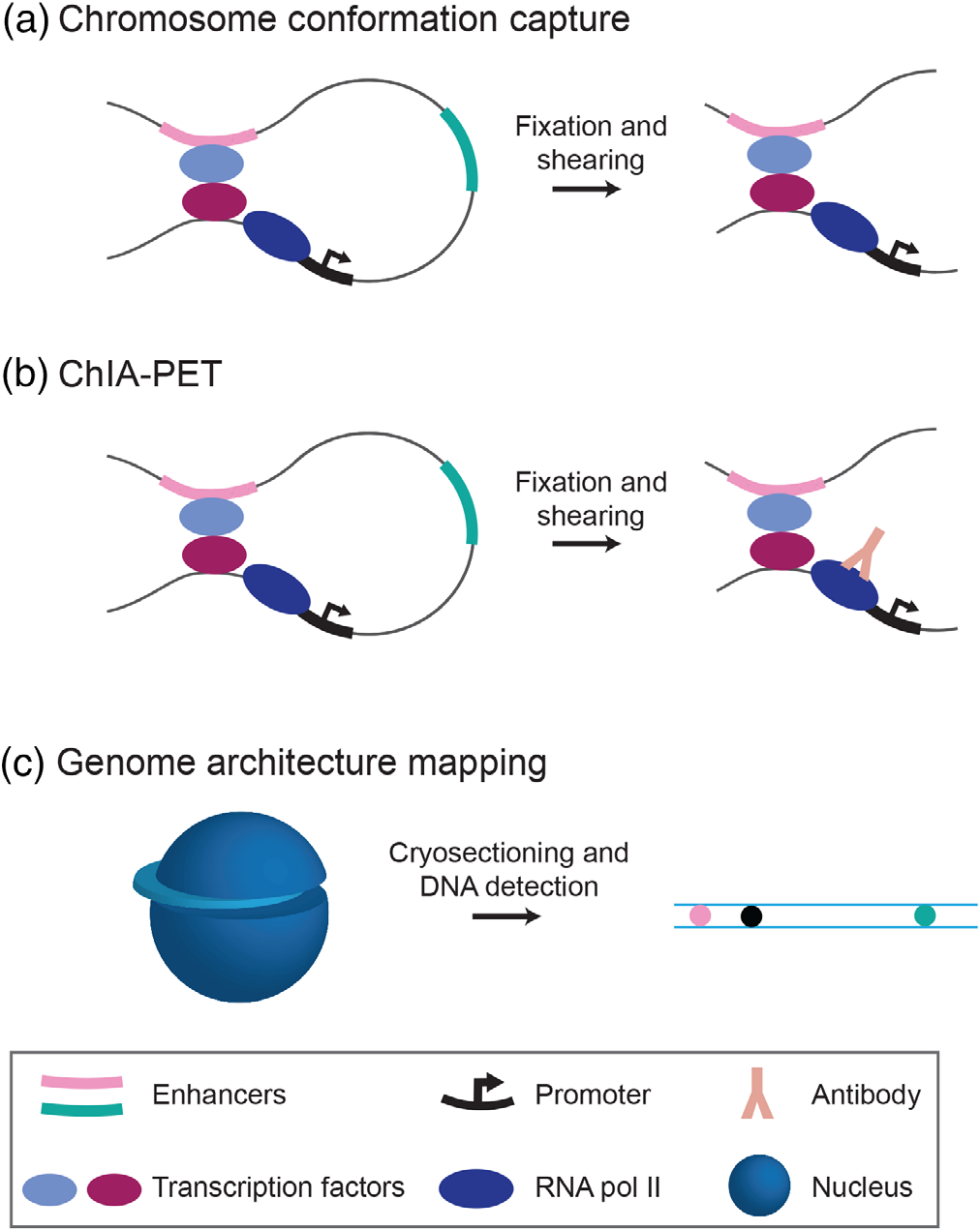
Genome‐wide methods to identify putative enhancer–promoter interactions. Distal enhancers (shown in pink) may be brought within close proximity to the promoters they regulate (shown in black) through the formation of chromatin loops. Looping is facilitated by protein–protein interactions of transcription factors that assemble at promoters and enhancers. The basic principles of methods to detect chromatin interactions are shown. Both (a) chromatin conformation capture (3C)‐based methods, including Hi‐C, and (b) chromatin interaction analysis with paired‐end tag sequencing (ChIA‐PET) preserve and detect chromatin interactions through crosslinking, fragmentation, and proximity ligation followed by high‐throughput sequencing. ChIA‐PET includes a chromatin immunoprecipitation step, often using antibodies targeting RNA pol II (shown), to enrich for complexes containing promoters. (c) Genome architecture mapping captures the distance between genomic loci by cryosectioning and laser microdissection (which allow spatial information to be preserved) followed by DNA sequencing

Enhancers can act over long distances, and are thought to be brought within close spatial proximity to the promoters they regulate. Thus, methods to study chromosomal interactions have also been used to identify putative enhancer–promoter interactions. The majority of these methods are based on chromosome conformation capture (3C) (Dekker, Rippe, Dekker, & Kleckner, 2002), in which chromatin is crosslinked and sheared, followed by proximity ligation reactions that capture physical chromosomal interactions (Figure 2a). The resulting DNA molecules contain regions that are not nearby in linear DNA sequence, but represent long‐range interactions. In 3C, specific interactions are assayed using PCR, but derivatives of this technique, including 4C and 5C, are higher throughput. The highest throughput of these methods is Hi‐C, in which proximity ligation is followed by massively parallel sequencing in order to capture all genomic interactions (Lieberman‐Aiden et al., 2009). Hi‐C was originally developed to investigate the spatial organization of the genome, which revealed the presence of topologically associating domains (TADs), regions of the genome that are highly self‐interacting (Dixon et al., 2012; Nora et al., 2012; Van Steensel & Dekker, 2010). Hi‐C has since been used to map genome‐wide interactions in numerous different organisms, tissues, and cell types, and has led to the identification of hundreds of thousands of putative enhancer–promoter contacts (Ron, Globerson, Moran, & Kaplan, 2017). The resolution of Hi‐C has improved from megabase resolution (Lieberman‐Aiden et al., 2009) of interactions to a few kilobases (kb) (Rao et al., 2014). However, this technique has not yet achieved sufficient resolution to detect enhancer–promoter interactions that take place on the hundred basepair scale.

A related strategy to identify genome‐wide interactions is chromatin interaction analysis with paired‐end tag sequencing (ChIA‐PET) (Figure 2b; Fullwood et al., 2009). This method pairs chromatin proximity ligation with ChIP to predict genomic interactions that include a protein of interest. ChIA‐PET typically utilizes antibodies against RNA polymerase II or TFs, which may enrich for functional interactions (Fullwood et al., 2009; G. Li et al., 2012). Advantageously, ChIA‐PET can generate genome‐wide chromatin interaction maps with a resolution of ~100 bp (Z. Tang et al., 2015). ChIA‐PET can also be used to probe putative enhancer–promoter interactions by pulling down proteins involved in these interactions, including cohesin and the mediator complex (Kagey et al., 2010; Schmidt et al., 2010; Whyte et al., 2013). Most recently, a droplet‐based method termed ChIA‐Drop has been developed to study complex chromatin interactions involving multiple loci (Zheng et al., 2019). This technique relies on microfluidics and barcode‐linked sequencing to map genomic interactions from crosslinked chromatin samples. ChIA‐Drop does not employ pairwise proximity ligations, and thus can capture multiplexed chromosomal contacts that may be useful for the study of enhancer–promoter interactions. However, this method is currently limited by its resolution of 5 to 10 kb, which is not sufficient to identify individual enhancer–promoter interactions (Zheng et al., 2019). Another method to study complex genomic interactions is genome architecture mapping (GAM) (Beagrie et al., 2017), which utilizes ultrathin cryosectioning and laser microdissection followed by DNA sequencing (Figure 2c). Within randomly oriented nuclear sections, loci in close physical proximity are found together more often than distant loci, allowing three‐dimensional maps of chromatin structure to be constructed. Unlike 3C‐based methods, GAM also captures chromatin compaction, radial distributions of chromosomes, and chromatin associations with the nuclear periphery (Beagrie et al., 2017). However, its current resolution of ~30 kb presently limits its utility in studying enhancer–promoter interactions.

### 2.6 Biological context matters in genome‐wide assays

Enhancers direct gene expression in specific cell types, at specific times, and within specific developmental contexts, and thus it is important to use appropriate and homogeneous samples when performing genome‐wide techniques to identify putative enhancers. For example, TFs and other proteins can be expressed in a highly tissue‐specific manner, which could affect ChIP‐seq experiments as they rely on a specific protein of interest being present. Furthermore, the chromatin landscape assayed by ATAC‐seq or Hi‐C can depend on the gene regulatory programs active in a given cell type. Indeed, chromatin interactions are highly dynamic and can vary substantially between different cell types (Schmitt et al., 2016). Heterogeneity in gene expression has even been observed in single cells from seemingly homogeneous starting material (Munsky, Neuert, & Van Oudenaarden, 2012; Shalek et al., 2013). Thus, when applied to tissues composed of diverse cell types, these methods can suffer from a low signal to noise ratio. This issue can potentially be overcome by single‐cell approaches, which have been developed for many of the genome‐wide methods described above, including ChIP‐seq (Rotem et al., 2015), CUT&RUN (Hainer, Bošković, McCannell, Rando, & Fazzio, 2019), CUT&TAG (Kaya‐okur et al., 2019), DNase‐seq (W. Jin et al., 2015), MNase‐seq (Lai et al., 2018), ATAC‐seq (Buenrostro et al., 2015; DeWitt et al., 2018), and Hi‐C (Nagano et al., 2013). Although highly scalable, at present the single‐cell versions of these methods yield sparse, low‐coverage data that are difficult to analyze using conventional approaches (Ji, Zhou, & Ji, 2017; Schep, Wu, Buenrostro, & Greenleaf, 2017). While sparsity of data is inherent to current single cell methods, advances in experimental and computational approaches may increase the resolution with which we can interrogate the genome for putative enhancers. Furthermore, improved single‐cell assays could be performed in each cell of a developing embryo to give unique insight into gene regulatory dynamics during development.

## 3 CORRELATIVE GENOME‐WIDE DATA CAN LEAD TO MISCONCEPTIONS

Genome‐wide assays have greatly advanced our ability to predict enhancer sequences, but we cannot accurately identify active enhancers without functional validation. Genome‐wide predictions of active enhancers are based on correlative data, and the presence of a given *trans*‐acting factor or chromatin feature does not mean that a given DNA element truly functions as an enhancer. Furthermore, these predictions of enhancer activity cannot determine which genes might be regulated by putative enhancers and do not assess any quantitative effects on gene expression. Without functional validation, findings from genome‐wide predictions of enhancer activity could lead to misconceptions. Currently, the most stringent validation of a predicted enhancer is to test its function in its native context in animal models. This approach is inherently low‐throughput, which significantly limits its practicality, since millions of candidate enhancers have been identified. However, several recent studies in animal models have demonstrated that there are indeed misconceptions in our understanding of how chromatin features relate to enhancer function, demonstrating the value of thorough functional validation to advance our understanding of enhancer function.

### 3.1 Misconceptions about super‐enhancer function

Genome‐wide mapping of features associated with active enhancers led to the concept of super‐enhancers, clusters of regulatory elements that span several kilobases (Hnisz et al., 2013; Lovén et al., 2013; Whyte et al., 2013). Super‐enhancers were first identified from ENCODE data as stretch enhancers or highly occupied target regions that are cell type‐specific and associated with increased expression of genes involved in cell type‐specific processes (Kurum, Benayoun, Malhotra, George, & Ucar, 2016; Kvon et al., 2012; Parker et al., 2013). Super‐enhancers also coincide with defined locus control regions (LCRs), genomic regions that encompass a set of regulatory elements and regulate the expression of one or more genes (Grosveld, van Assendelft, Greaves, & Kollias, 1987; Q. Li, Peterson, Fang, & Stamatoyannopoulos, 2002; Pott & Lieb, 2015). Super‐enhancers are further characterized by binding of the mediator complex and H3K27ac, and are frequently flanked by insulator elements (Ing‐Simmons et al., 2015; Whyte et al., 2013). It was also observed that super‐enhancer‐associated genes are expressed at higher levels than genes associated with typical enhancers, and that super‐enhancers are highly sensitive to perturbation (Hnisz et al., 2013; Lovén et al., 2013; Whyte et al., 2013). These findings led to the idea that super‐enhancers may be more than the sum of their parts and represent a new paradigm in gene regulation. However, super‐enhancers as a class were defined based on these features without being functionally validated. Thus, it remains unclear whether they represent a simple clustering of conventional enhancers, or whether the constituent elements of a super‐enhancer may act together to produce novel and synergistic functional properties.

A cluster of regulatory elements constituting a super‐enhancer controls expression of the α‐globin gene in mouse erythroid cells (Hay et al., 2016). Each of the five individual elements in this super‐enhancer scored as an erythroid enhancer based on chromatin signatures and transient reporter assays. However, three of these elements failed to drive expression in a classic mouse transgenic assay, in which a construct containing an enhancer, minimal promoter, and lacZ reporter is stably inserted into the mouse genome (Hay et al., 2016). The authors of this study next dissected the function of this proposed super‐enhancer by generating several mouse models in which each constituent enhancer was deleted, individually or in pairs (Hay et al., 2016). Mice with homozygous deletion of any one of these enhancers were viable, had normal steady‐state levels of α‐globin mRNA, and did not exhibit any overt phenotypes (Hay et al., 2016). Two of the five enhancers were characterized by high levels of erythroid TF binding in ChIP‐seq assays, exhibited the strongest enhancer activity, and were embryonic lethal when deleted in combination. Deletion of either of these two strong enhancers caused stress erythropoiesis, which ensures normal hemoglobin production, suggesting that multiple enhancers at this locus ensure robust gene expression.

Altogether, these experiments demonstrated that components of the α‐globin super‐enhancer contribute to gene expression in an additive rather than synergistic manner, challenging the notion that super‐enhancers are a distinct class of regulatory element. However, another study found non‐additive effects within a mammary gland‐specific super‐enhancer of *Wap* (Shin et al., 2016). At present, few super‐enhancers have been rigorously tested and the functional differences between super‐enhancers and clusters of enhancers, if any, remain unclear (Pott & Lieb, 2015). Nevertheless, it has been known for decades that clusters of enhancers commonly regulate developmental genes, and thus it will be crucial to continue to functionally validate these clusters of enhancers identified from genome‐wide data. Indeed, the concept of super‐enhancers remains an important area of investigation, and continues to be used to describe highly active, cell type‐specific loci in development and disease.

### 3.2 Misconceptions about the importance of histone modifications associated with enhancers

While histone modifications are useful for identifying regions within the genome that may contain enhancers, these epigenetic marks are not necessarily required for enhancer function. H3K4me1 is a highly conserved chromatin feature associated with active enhancers, and is catalyzed by the complex of proteins associated with Set1 (COMPASS)‐like methyltransferase family (Creyghton et al., 2010; Heintzman et al., 2007). In *Drosophila*, this family includes the methyltransferase Trr, which is essential for successful development (Herz et al., 2012; Shilatifard, 2012). However, the requirement of enzymatically active Trr for successful development was more recently tested. Intriguingly, it was found that *Drosophila* embryos expressing catalytically inactive Trr, and thus lacking H3K4me1 marks, exhibit only mild phenotypes and develop to productive adulthood (Dorsett et al., 2017). Similarly, flies expressing a hyperactive Trr allele that changes the enzyme product specificity were viable and exhibited only subtle phenotypic effects, despite H3K4me1 being converted to H3K4me2 or H3K4me3 (Dorsett et al., 2017). Gene expression was largely unchanged in these mutant *Drosophila* lines, although diminished H3K4me1 at enhancers was associated with decreased expression of the nearest gene. Nonetheless, this study demonstrated that global loss of a conserved chromatin feature associated with active enhancers is compatible with life, challenging the idea that H3K4me1 is required for successful development. Thus, H3K4me1 appears to be only correlative with, rather than causative of, enhancer function. Moving forward, it will be important to investigate whether other epigenetic marks associated with enhancers have functional roles in gene regulation and development.

### 3.3 Misconceptions about topologically associated domains

The appearance of TADs revealed by Hi‐C and related methods promised to give insight into the relationship between genome architecture and gene regulation. A handful of studies found that changes in chromosome structure that affect TAD boundaries were associated with aberrant enhancer activity and gene expression (Blinka, Reimer, Pulakanti, & Rao, 2016; Franke et al., 2016; Lupiáñez et al., 2015). Developmental genes and their enhancers are commonly found within the same TAD (Williamson, Lettice, Hill, & Bickmore, 2016), and TAD boundaries tend to be demarcated with binding sites for the CCCTC‐binding factor (CTCF; Dixon et al., 2012; Phillips‐Cremins et al., 2013), which can exhibit insulator activity (Ghirlando & Felsenfeld, 2016; Merkenschlager & Nora, 2016). Together, these observations led to a model in which TAD boundaries act as insulators that prevent enhancers from regulating genes outside of their topological domains (Doyle, Fudenberg, Imakaev, & Mirny, 2014; Symmons et al., 2014). However, functional investigation of TADs has called into question the role of TADs in gene regulation. For example, global disruption of TADs by deletion of a cohesin‐loading factor has no effect on transcriptional activity (Schwarzer et al., 2017). Furthermore, several recent studies have reported that depletion of CTCF at TAD boundaries does not impact gene expression (Despang et al., 2019; Gambetta & Furlong, 2018; Kubo et al., 2017; Soshnikova, Montavon, Leleu, Galjart, & Duboule, 2010) histone modifications (Kubo et al., 2017), or even TAD structure (Kubo et al., 2017; Zuin et al., 2014).

The roles of CTCF and TAD boundaries in developmental gene regulation were recently studied further using the sonic hedgehog (Shh) locus as a model system (Williamson et al., 2019). Shh is located within a TAD that contains all of its enhancers, including the ZRS enhancer that drives restricted expression in the developing limb bud (Lettice et al., 2003). In mouse, changing the TAD structure by deleting CTCF sites or creating large inversions or deletions remarkably had no effect on *Shh* patterning or phenotype, despite increased distance between the Shh promoter and the ZRS by fluorescent in situ hybridization (FISH) (Williamson et al., 2019). A recent study in *Drosophila* utilized flies carrying highly rearranged balancer chromosomes to measure allele‐specific changes in chromatin topology and gene expression (Ghavi‐Helm et al., 2019). Rearrangement or fusing of TADs, or disruption of long‐range chromatin loops generally did not correlate with changes in gene expression, despite covering 75% of the *Drosophila* genome. However, gene expression at a few distinct loci did appear to be sensitive to TAD perturbations (Ghavi‐Helm et al., 2019). Indeed, another recent study found that the human endogenous retrovirus subfamily H (HERV‐H) has a role in establishing TAD boundaries in human pluripotent stem cells, and that deletion of HERV‐H elements disrupts their corresponding TADs and reduces the transcription of nearby genes (Zhang et al., 2019). While still controversial, there is a growing consensus that TADs do not globally mark regulatory boundaries in the genome. Instead, recent evidence suggests that organization of the genome into TADs may play a role in DNA replication (Jodkowska et al., 2019; Pope et al., 2014).

### 3.4 Misconceptions about chromatin looping

Another recent study of the Shh locus highlighted that we still do not understand precisely how enhancers interact with and activate transcription from promoters. Indeed, there are likely several different mechanisms through which enhancers and promoters interact to regulate gene expression. The physical distance between Shh and a distal brain enhancer increases rather than decreases upon enhancer activation, challenging the model that distal enhancers must interact with promoters through chromatin looping (Benabdallah et al., 2017). Instead, it appears that activation of this brain enhancer induces large‐scale chromatin decompaction, suggesting a new mechanism of long‐range gene regulation (Benabdallah et al., 2017).

Chromatin looping has been clearly illustrated between Shh and the ZRS (Symmons et al., 2016; Williamson et al., 2016), and between the beta‐globin gene and its distal LCR enhancer (Carter, Chakalova, Osborne, Dai, & Fraser, 2002; Tolhuis, Palstra, Splinter, Grosveld, & De Laat, 2002). Furthermore, in *Drosophila* the sex combs reduced (Scr) promoter is known to be brought into contact with its distal T1 enhancer by a promoter‐proximal tethering element (Calhoun, Stathopoulos, & Levine, 2002). It is presently unknown how many enhancer–promoter interactions are mediated by tethering elements and what other mechanisms may be involved. Indeed, only a handful of enhancer–promoter interactions have been thoroughly studied, and thus it remains unclear how pervasive chromatin looping between promoters and distal enhancers is as a gene regulatory mechanism. An emerging model of enhancer–promoter interaction posits that transcriptional control may be driven by the formation of condensates or hubs that concentrate the transcriptional machinery (Banani, Lee, Hyman, & Rosen, 2017; Boija et al., 2018; Hnisz, Shrinivas, Young, Chakraborty, & Sharp, 2017).

Condensation of TFs and cofactors is thought to be mediated by the low‐complexity, intrinsically disordered regions of their activation domains, and can result in droplets that exhibit properties of liquid–liquid phase separation (Cho et al., 2018; Chong et al., 2018; Sabari et al., 2018). Phase‐separated condensates incorporate many factors known to associate with enhancers, including the mediator complex, and may be particularly prevalent at clusters of enhancers to promote efficient activation of gene expression (Cho et al., 2018; Sabari et al., 2018). Furthermore, it has been suggested that condensates may contribute to long‐range enhancer–promoter interactions as well as higher‐order genome organization (Shrinivas et al., 2019). However, we currently lack a clear understanding of what functional properties phase separation may confer, and how phase separation should be defined and experimentally validated in biological systems (Mir, Bickmore, Furlong, & Narlikar, 2019). Indeed, several recent studies have highlighted transcriptional hubs that resemble phase‐separated condensates but appear to be driven by independent mechanisms (McSwiggen et al., 2019; Mir et al., 2018). At present, it remains unclear whether phase separation has functional roles in gene regulation, or whether the appearance of phase‐separated condensates is simply a consequence of transient protein–protein interactions (Chong et al., 2018).

While there is certainly more work to be done to work out general principles of gene regulation, genome‐wide maps of chromosomal contacts have provided a wealth of candidate enhancer–promoter interactions. When using chromatin interaction data to identify candidate enhancers, it is important to remember that physical contact between two genomic loci does not necessarily mean that a causal regulatory relationship exists. Furthermore, direct contact between a distal enhancer and promoter may not be required for transcriptional activation or successful development. For example, a recent study used circularized chromosome conformation capture (4C) to characterize the regulatory topology of Pitx1, which is required for hindlimb and mandibular arch development (Sarro et al., 2018). 4C identified a robust, hindlimb‐specific interaction between the Pitx1 promoter and a distal, putative hindlimb enhancer. Deletion of this putative enhancer in mouse completely disrupted the 4C interaction, but hindlimb expression of *Pitx1* was only mildly affected; furthermore, the mice did not exhibit any developmental defects typical of Pitx1 gene deletion (Sarro et al., 2018). Although *Pitx1* was still expressed in mice lacking this enhancer, 4C did not detect any compensatory interactions with the Pitx1 promoter, suggesting that gene expression may be activated by other enhancers in the absence of direct enhancer–promoter interactions. However, it is also possible that this finding may be due to limitations in the resolution of the assay (Raviram, Rocha, Bonneau, & Skok, 2014). Nevertheless, the results of this study demonstrate that tissue‐specific physical interactions involving essential developmental genes may have limited predictive power in identifying enhancers that are required for proper development and highlight the complex nature of gene regulation during development.

Like other genome‐wide methodologies used to infer active enhancer sequences, we can make better predictions when chromatin interaction data are paired with additional evidence. A recent study used this approach to identify putative causal variants for blood cell traits from genome‐wide association study (GWAS) data (Lareau et al., 2018). Fine‐mapped variants were overlaid with ATAC‐seq data to identify those that might have a role in gene regulation, and from this list ChIP‐seq datasets revealed variants that might disrupt TF binding motifs. Hi‐C data was then used to identify the putative target genes of regulatory variants. Altogether, these complementary strategies reduced the number of putative causal variants 100‐fold, providing a reasonable number of candidates that could be directly tested in functional assays. Given the vast number of putative enhancers that have been identified, combining these approaches is a viable strategy to prioritize putative regulatory elements for functional validation.

### 3.5 Validation is key to understanding enhancer function

Genome‐wide assays have revolutionized our ability to study the *cis*‐regulatory genome, but making assumptions from these data without functional validation has led to misconceptions. Such misconceptions could impeed our ability to understand the mechanisms through which enhancers regulate gene expression and prevent us from elucidating which enhancer sequences are important in development and disease processes. The studies highlighted above underscore the importance of functionally validating putative enhancers. Altogether, they provide new insight into the requirements of different chromatin features for enhancer function and successful development, and suggest that epigenetic marks are associated with enhancers rather than causative of enhancer function. Although inherently low‐throughput, transgenic animal models remain an important tool in the dissection of *cis*‐regulatory sequences, and highlight the need for complementary approaches to identify functional enhancers.

## 4 MASSIVELY PARALLEL REPORTER ASSAYS TO STUDY ENHANCER FUNCTION

Chromatin‐state mapping has identified myriad candidate enhancers across the human genome; however, these approaches are primarily descriptive and may not necessarily inform enhancer function. Furthermore, they cannot explain why a given *cis‐*regulatory element is active in a particular cell type, or predict the effects of specific sequence changes within an enhancer. Classic mouse transgenic approaches are a useful tool with which to test the necessity or sufficiency of individual enhancer sequences for a given biological outcome, but are highly impractical for validating putative enhancers at scale.

**Figure 3:**
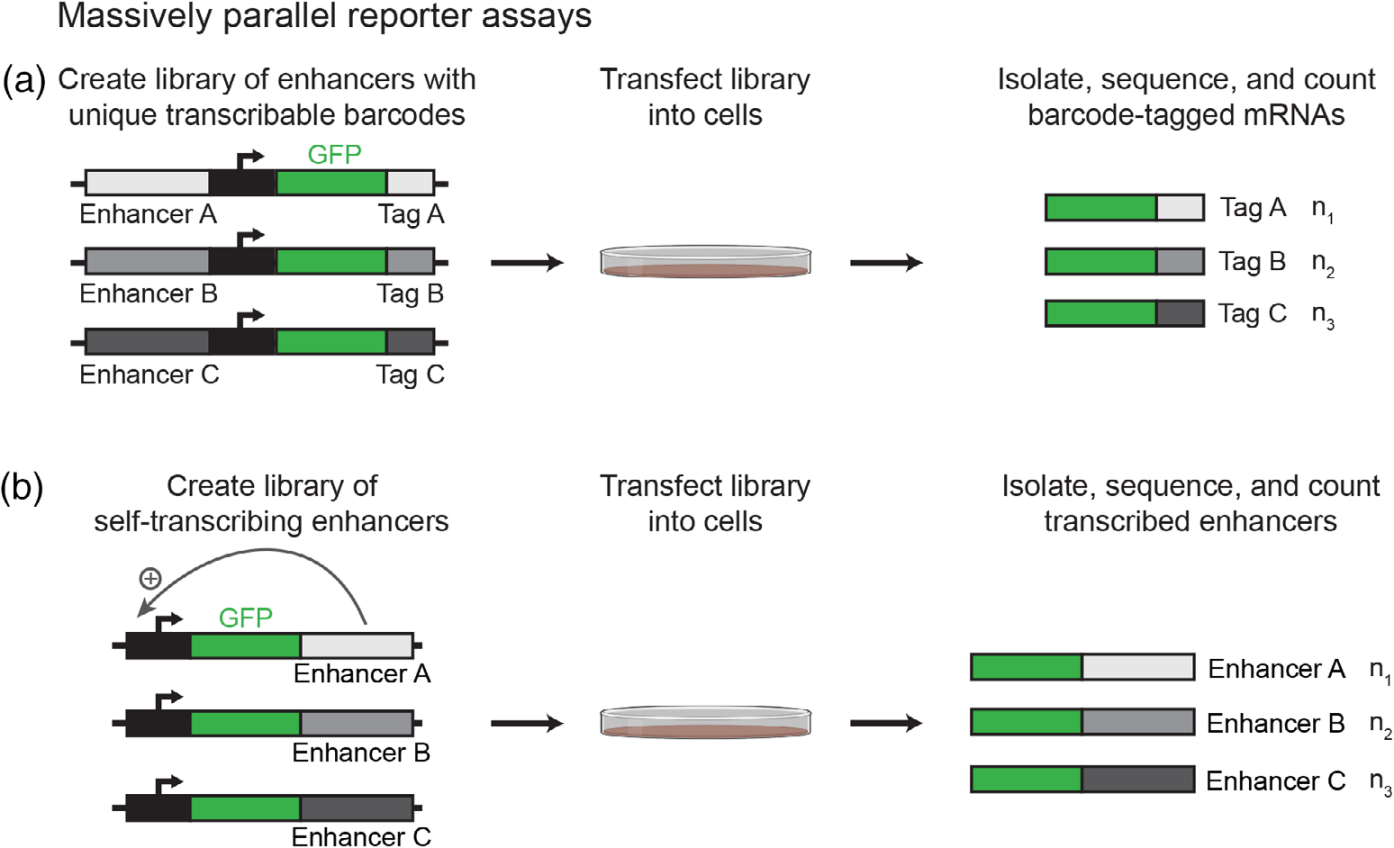
In vitro massively parallel reporter assays (MPRAs) to study enhancer function. MPRAs utilize advances in oligonucleotide synthesis and sequencing technology to test large libraries of candidate enhancer sequences for function. (a) MPRA libraries typically contain candidate enhancer sequences upstream of a minimal promoter and reporter gene. A transcribable barcode unique to each candidate enhancer is placed in the 3′ untranslated region of the reporter gene, allowing active enhancers to be identified by sequencing barcode‐tagged mRNAs. (b) Self‐transcribing active regulatory region sequencing (STARR‐seq) is a variation of the MPRA that exploits the characteristic that enhancers can function independently of their relative positions. In STARR‐seq, candidate enhancer sequences are placed downstream of a minimal promoter and reporter gene, allowing active enhancers to transcribe themselves. Figure created with BioRender

To overcome bottlenecks in the functional validation of candidate enhancer sequences identified by genome‐wide methods, massively parallel reporter assays (MPRAs) have been developed. These methods are based on traditional reporter assays, in which a putative regulatory element is placed upstream of a reporter coding sequence (e.g., luciferase) in a vector, transfected into cells, and assayed for reporter activity. Although the context is often heterologous, that is, using genetic elements from other organisms, these assays are a powerful tool with which to test the sufficiency of an element to regulate gene expression. MPRAs utilize advances in oligonucleotide synthesis (LeProust et al., 2010) and sequencing technology to expand upon this experimental framework. In MPRAs, libraries containing hundreds of thousands of candidate enhancers can be generated and tested for function in parallel (Figure 3a). Each enhancer is cloned upstream of a minimal promoter and a reporter gene containing a unique sequence barcode in its 3′ untranslated region. These libraries of barcoded reporter genes can then be introduced into cells or animals and expression quantified by RNA‐seq of the unique sequence barcodes. Thus, MPRAs can be used to functionally validate numerous putative enhancer sequences in a single experiment with a quantitative readout. Furthermore, because so many sequences can be tested for function at once, MPRAs enable the study of how single nucleotide changes within an enhancer affect function. This systematic dissection of enhancer sequences has given important insight into the mechanistic basis of enhancer activity and the functional constraints of enhancer sequences during development.

### 4.1 MPRA approaches to study enhancer function

MPRAs have successfully been utilized in a number of diverse biological systems to (a) interrogate the activity of candidate enhancer sequences and (b) to investigate the functional consequences of mutations within known enhancers. Several studies have used MPRAs to functionally validate putative enhancers identified from genome‐wide assays (Kheradpour et al., 2013; Kwasnieski, Fiore, Chaudhari, & Cohen, 2014). To study enhancer sequence variation, one MPRA report used the human embryonic kidney cell line HEK293T to study all possible single nucleotide variants of two inducible enhancers, a synthetic cAMP‐regulated enhancer and a virus‐induced enhancer of *IFNB* (Melnikov et al., 2012). The authors of this study used their data from induced versus uninduced cellular states to generate a model that could predict the activity of novel variants. This allowed for the design of synthetic enhancers that optimize different objectives, for example, minimizing basal activity while maximizing induced activity (Melnikov et al., 2012), and thus this method has important implications for synthetic biology.

In another MPRA saturation mutagenesis approach, three mammalian liver enhancers were dissected at single‐nucleotide resolution by injecting libraries containing hundreds of thousands of mutant enhancer sequences into mouse tail vein (Patwardhan et al., 2012). Following injection, livers were harvested and transcribed barcodes were sequenced. This study found that only a small fraction of enhancer mutations affected activity by more than twofold, and sequence changes with higher effects tended to fall within known liver TF binding sites (Patwardhan et al., 2012). In contrast to these modest effects, another saturation mutagenesis MPRA study found that the majority of sequence variants tested within a *Rhodopsin* promoter proximal enhancer had significant effects on regulatory activity in mouse retina (Mogno, Kwasnieski, Cohen, Myers, & Corbo, 2012). Not all of these effects could be explained by changes in the affinity of known TF binding sites. The different results obtained by these two studies likely reflect differences in the specific enhancers examined, study design, and biological system utilized. Indeed, an MPRA that analyzed exonic liver enhancers found different effects of enhancer mutations in liver compared to HeLa cells (Birnbaum et al., 2014), highlighting the importance of context‐specific MPRA approaches.

An alternate MPRA approach has been developed, termed self‐transcribing active regulatory region sequencing (STARR‐seq; Figure 3b; Arnold et al., 2013; Schöne et al., 2018). This method employs the characteristic that enhancers can function independently of their relative positions, and places candidate sequences downstream of a minimal promoter and open reading frame. Thus, in this system active enhancers transcribe themselves, negating the need for barcode sequences. A STARR‐seq library containing millions of randomly sheared genomic DNA fragments from *Drosophila* were transfected into S2 cells followed by RNA‐sequencing to identify transcribed elements (Arnold et al., 2013). This study identified thousands of genomic regions that were significantly enriched with a wide range of enhancer activity, with the strongest enhancers found next to housekeeping genes and developmental TFs. This approach also revealed that 31% of strong enhancers in S2 cells were located within closed chromatin and lacked H3K27ac, but were marked with H3K4me1, suggesting that these sequences may be silenced in their endogenous contexts. This finding highlights the utility of MPRAs as a complementary approach to genome‐wide chromatin‐state assays. Like other MPRAs, STARR‐seq is easily adaptable to the study of synthetic enhancer variants (Schöne et al., 2018).

### 4.2 MPRAs to study enhancer function in whole developing embryos

**Figure 4:**
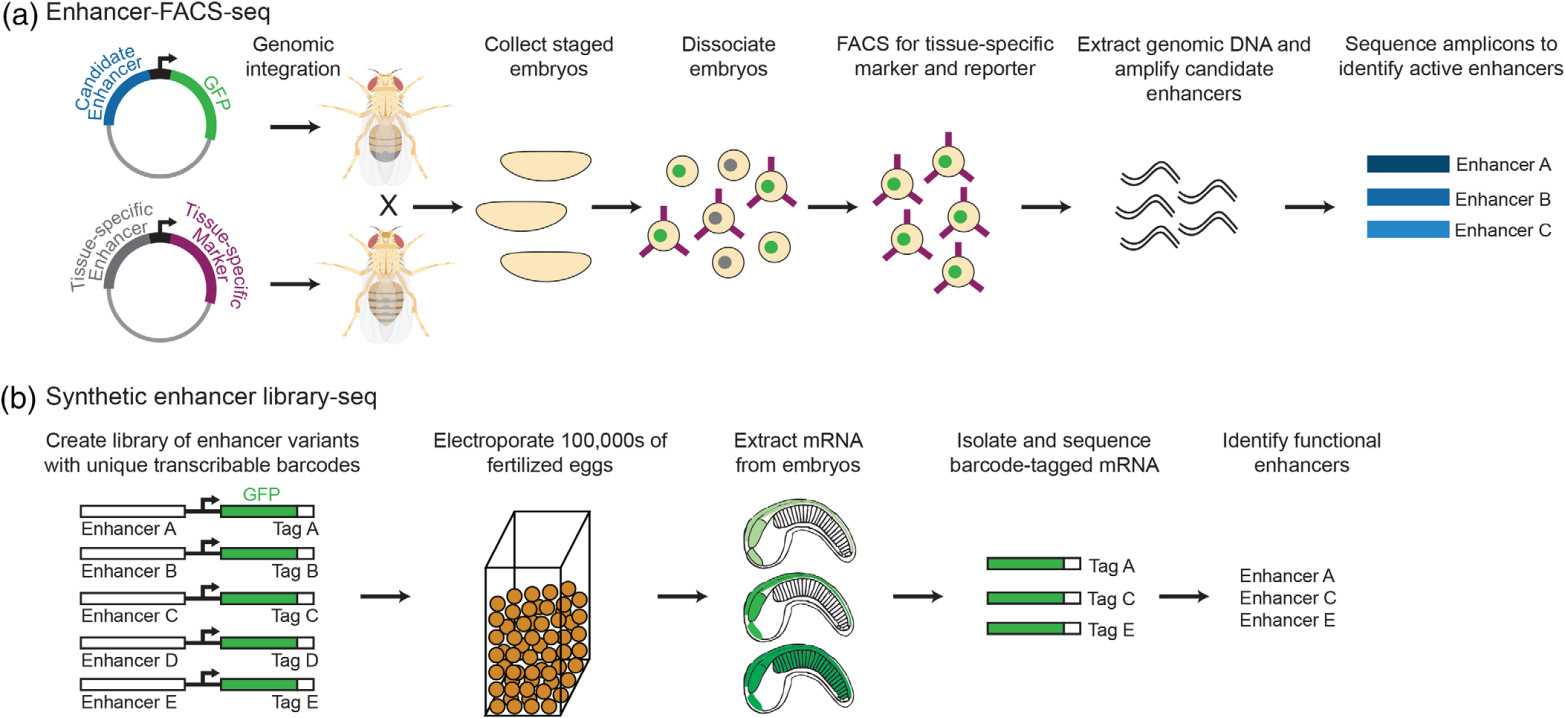
In vivo massively parallel reporter assays to study enhancer function. (a) Enhancer‐FACS‐seq identifies active, tissue‐specific enhancers in whole *Drosophila* embryos. Two flies are crossed, the first carrying a candidate enhancer sequence driving GFP expression, and the second expressing a cell surface marker in a tissue‐specific manner. Embryos resulting from this cross are dissociated and FACS sorted for the tissue‐specific cell surface marker and GFP. Genomic DNA can then be extracted from sorted cells to identify enhancers that are active in the specific tissue. (b) Synthetic enhancer library‐sequencing (SEL‐seq) allows millions of enhancer variants to be tested for function in whole *Ciona* embryos. Synthetic enhancer variants are attached to a minimal promoter, GFP coding sequence, and a unique transcribable barcode. The enhancer library is electroporated into hundreds of thousands of fertilized *Ciona* eggs, which are then allowed to develop until the desired developmental stage. Barcode‐tagged mRNA can then be isolated and sequenced to identify active enhancer variants, providing insight into which sequences within an enhancer are important for function. Figure created with BioRender

MPRA methodologies have also been adapted for use in whole developing embryos, providing novel insight into the function and tissue‐specificity of developmental enhancers. Enhancer‐FACS‐seq (eFS) was developed in *Drosophila* to identify active, tissue‐specific enhancers in whole embryos (Figure 4a; Gisselbrecht et al., 2013). This method utilizes a cross between two transgenic fly lines, one carrying a cassette containing a candidate enhancer upstream of a reporter gene and the other expressing a cell‐surface antigen in a tissue‐specific manner. The candidate enhancer‐reporter construct is integrated into the genome in a site‐specific manner to avoid position effects. A cross between these fly strains generates embryos that can be dissociated and FACS sorted, first by tissue type and then by reporter expression. Genomic DNA can then be isolated from the collected cells and sequenced to identify enhancers activated in specific tissues. eFS was first used to test hundreds of putative enhancer sequences for function in whole mesoderm, fusion‐competent myoblasts, and somatic mesoderm founder cells of developing *Drosophila* embryos. This identified over 100 active mesoderm enhancers that correlated well with histone marks of active enhancers and were enriched for RNA polymerase II and mesoderm‐specific TF binding by ChIP‐seq (Gisselbrecht et al., 2013).

Because it is not feasible to test all enhancers in all tissues at all developmental timepoints, identifying which sequences within an enhancer are important for function would allow us to develop a set of rules that define how enhancers drive tissue‐specific expression. These rules could then be used to better identify enhancers in the genome prior to functional validation. To understand how enhancer sequences encode function, whole‐embryo MPRAs were developed in the tunicate *Ciona intestinalis*, which belongs to the sister group of all vertebrates (Delsuc, Brinkmann, Chourrout, & Philippe, 2006). Developmental programs are conserved between *Ciona* and vertebrates, and thus findings in *Ciona* are expected to be highly relevant to the study of enhancer function during vertebrate development. The synthetic enhancer library‐sequencing (SEL‐seq) method is able to test millions of enhancer variants for function in developing *Ciona* embryos (Figure 4b). Millions of *Ciona* embryos can be electroporated with reporter constructs in a single experiment, making it highly amenable to testing enhancers at scale.

SEL‐seq was first used to study a minimal, 69‐basepair Orthodenticle homeobox (*Otx*) enhancer that contains GATA and ETS binding sites and directs specific expression in the neural plate (Bertrand, Hudson, Caillol, Popovici, & Lemaire, 2003; Farley et al., 2015; Rothbacher, Bertrand, Lamy, & Lemaire, 2007). Millions of synthetic enhancers were created in which core GATA and ETS binding motifs remained constant and the remaining sequence was randomized (Farley et al., 2015). Each synthetic enhancer is attached to a minimal promoter, GFP coding sequence, and unique barcode sequence. The Otx enhancer library was electroporated into fertilized *Ciona* eggs, which developed to the stage at which the wildtype Otx enhancer becomes active and were then harvested for RNA. RNA‐seq of transcribed barcodes allowed active enhancers to be identified. Computational analysis of ~20,000 highly active enhancer variants revealed that dinucleotide motifs flanking core ETS and GATA binding sites are key determinants of enhancer function (Farley et al., 2015).

When the wildtype dinucleotide motifs were added to an otherwise inactive enhancer variant, the wildtype expression pattern was recapitulated. When these dinucleotide motifs were optimized to match the highest affinity position–weight matrices identified by in vitro binding assays, the previously inactive synthetic enhancer drove robust, ectopic expression. The strength and specificity of enhancer sequences could be further modulated by changing the spacing and orientation of TF binding sites (Farley et al., 2015; Farley, Levine, et al., 2016). The SEL‐seq method revealed that suboptimal affinity and spacing of GATA and ETS motifs within the Otx enhancer are important for tissue‐specific expression, and suggested a tradeoff between TF binding site affinity and syntax within developmental enhancers. Furthermore, analysis of ectopically expressed enhancer variants led to the identification of a novel notochord enhancer and demonstrated enhancer grammar, the interplay between TF binding site affinity and organization (Farley, Levine, et al., 2016). Defining the grammatical constraints for notochord enhancers enabled the identification of notochord‐specific enhancers within the genome, demonstrating the value of understanding grammatical constraints on enhancer function. Altogether, high‐throughput reporter assays in *Ciona* have made substantial progress toward elucidating the regulatory principles governing enhancer function.

### 4.3 Current limitations of MPRAs

MPRAs have greatly increased our ability to test the sufficiency of candidate enhancer sequences for expression at scale, and have proven to be a powerful method with which to study how variation within enhancer sequences impacts function. This has allowed us to gain significant insight into which parts of an endogenous enhancer sequence are important for function. However, there are several important considerations to be made when designing MPRAs and interpreting their results. For example, it is critical to use a minimal promoter that does not drive expression in the absence of enhancer sequences, as basal promoter activity could confound results. Furthermore, enhancer activity can vary substantially in different promoter contexts (Zabidi et al., 2015). Current limitations on the length of synthesized oligos makes it difficult to comprehensively study variation within enhancer sequences greater than 200 basepairs (Kwasnieski et al., 2014; Tewhey et al., 2016). Additionally, most MPRAs thus far have been done in cell lines which may not recapitulate the in vivo function of enhancers and limits their insight into development (Erceg et al., 2014). This limitation has been addressed by recent MPRA approaches in whole embryos, which have begun to reveal how enhancer sequences encode tissue‐specific expression during development (Farley et al., 2015; Gisselbrecht et al., 2013). Finally, MPRAs typically utilize heterologous reporter constructs that test enhancer sequences for function outside of their native genomic contexts. This potential limitation of MPRAs has recently been addressed by genome editing technology. However, manipulating enhancer sequences within the genome may produce modest phenotypes due to enhancer redundancy, or conversely, interfere with development, making it difficult to observe normal expression patterns. It is thus important to employ both MPRA and genome editing approaches to gain a comprehensive understanding of enhancer function. Indeed, MPRAs remain an essential and complementary tool because they allow enhancer sequences to be directly and systematically tested for function.

## 5 CRISPR/CAS9‐BASED METHODS TO IDENTIFY CANDIDATE ENHANCERS

**Figure 5:**
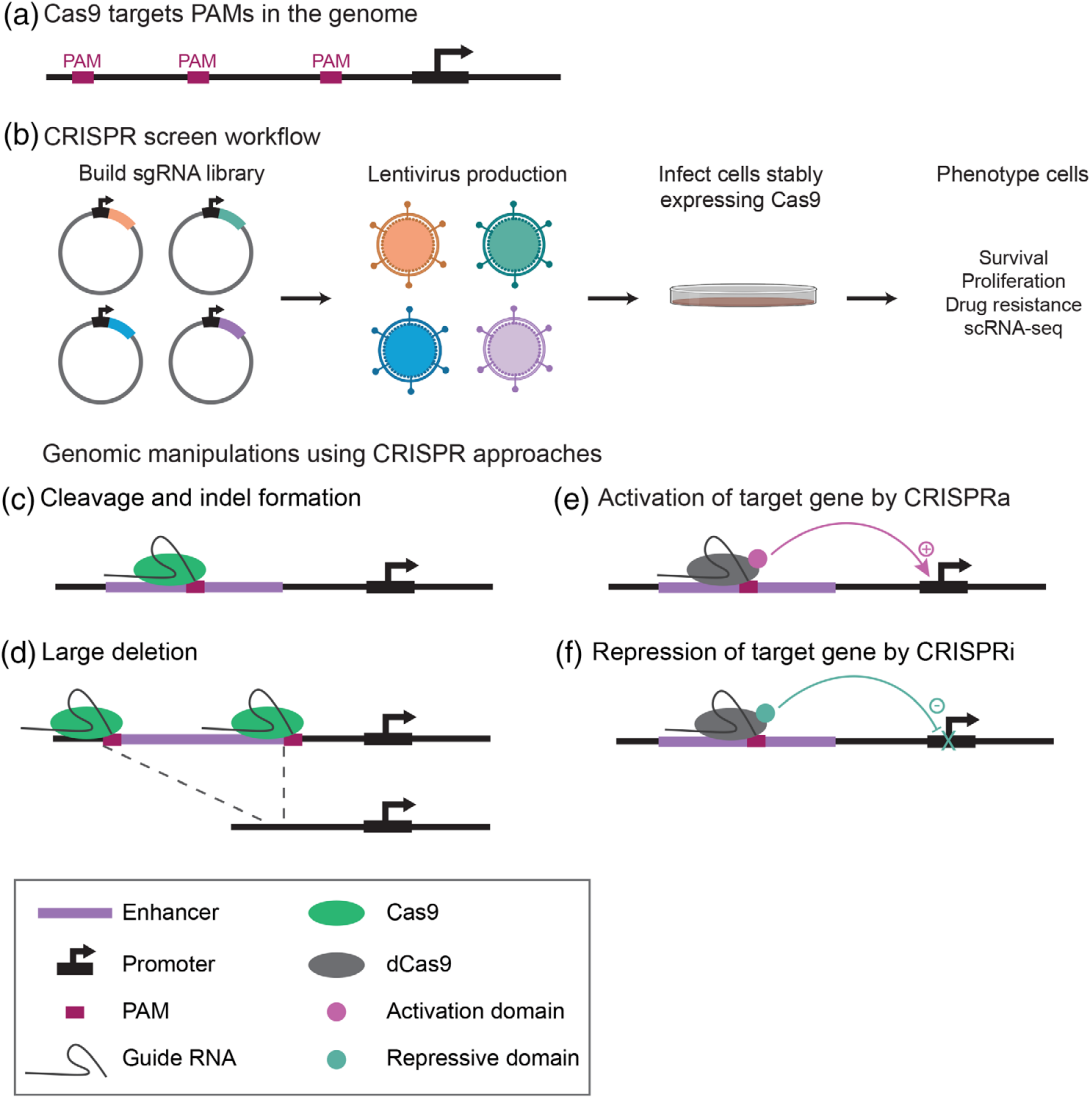
CRISPR‐based methods to identify and study candidate enhancers. (a) Any genomic region containing a protospacer‐adjacent motif (PAM) can be targeted by CRISPR/Cas9. (b) The workflow for high‐throughput CRISPR screens includes building a library of sgRNAs, cloning the library into lentiviral vectors, infecting cells that stably express Cas9 or one of its variant forms, and phenotyping cells. The sgRNA(s) carried by cells of a given phenotype can easily be identified by sequencing, and can give insight into candidate *cis‐*regulatory regions that affect phenotype. CRISPR screens can utilize (c) one sgRNA to mutate a particular locus, or (d) two sgRNAs to delete a genomic region. In both cases, DNA cut by Cas9 will be repaired by the error‐prone non‐homologous end joining (NHEJ) pathway, creating indels. Variant forms of Cas9 that are used in high‐throughput screens include (e) CRISPRa to activate transcription and (f) CRISPRi to repress transcription. Both CRISPRa and CRISPRi utilize catalytically dead Cas9 fused to an effector protein and impact gene expression through chromatin remodeling. Figure created with BioRender

The development of genome editing technology using Clustered Regularly Interspaced Short Palindromic Repeats (CRISPR) and the CRISPR‐associated protein 9 (Cas9) (Cong et al., 2013; Mali et al., 2013) has revolutionized our ability to interrogate putative enhancer sequences for function within their native genomic context. In this system, the Cas9 nuclease is guided by a single guide RNA (sgRNA; Jinek et al., 2012) to a specific DNA target, where it induces a double strand break (DSB). Cas9‐induced DSBs are typically repaired by non‐homologous end joining (NHEJ), an error‐prone DNA repair pathway that creates random insertion or deletion mutations (indels) at the site of cleavage. CRISPR/Cas9 is advantageous over traditional genetic manipulations because it can target any region of the genome containing a protospacer‐adjacent motif (PAM) (Figure 5a) and has been shown to effectively edit the genomes of diverse organisms (Friedland et al., 2013; Nakayama et al., 2013; Shan et al., 2013; Stolfi, Gandhi, Salek, & Christiaen, 2014; Wang et al., 2013; Yang et al., 2014; Yu et al., 2013). Furthermore, editing by CRISPR/Cas9 has improved efficiency compared to traditional reverse genetics approaches (Burgio, 2018). Studying putative *cis‐*regulatory elements individually can be extremely informative and is currently the standard for functional validation. However, due to the large number of putative enhancer sequences identified by genome‐wide assays, a major focus has been to develop massively parallel cell‐based CRISPR/Cas9 screens to functionally test the effects of *cis*‐regulatory changes on gene expression and phenotype. While this cutting‐edge technology has not yet been applied to developing embryos, advances in high‐throughput CRISPR screens could provide unprecedented insight into the regulation of gene expression during development.

### 5.1 High‐throughput CRISPR screens to identify candidate enhancers

To identify candidate enhancer sequences, high‐throughput CRISPR/Cas9 screens typically utilize tens of thousands of sgRNAs targeting the noncoding genome, which are cloned into lentiviral vectors and delivered as a pool to cells at a low multiplicity of infection (MOI) along with Cas9 (Figure 5b). The use of a low MOI ensures that, on average, cells are infected with only one sgRNA construct and thus bear only one CRISPR‐induced mutation (Figure 5c). Cells are then selected for a phenotype, such as survival, proliferation, or drug resistance, and sgRNAs that are enriched or depleted in cells give insight into putative *cis*‐regulatory regions that affect phenotype. Findings from these pooled screens can then be prioritized and validated in follow‐up experiments using individual sgRNAs.

High‐throughput CRISPR/Cas9 screens (Canver et al., 2015; Gasperini et al., 2017; Korkmaz et al., 2016; Sanjana et al., 2016; Shen et al., 2017) have been used to identify putative enhancer sequences by (a) mutating the sequence proximal to a specific gene or (b) mutating the binding sites of a specific TF genome‐wide. For example, a pooled CRISPR screen was used for saturating mutagenesis across an erythroid lineage‐specific enhancer associated with hemoglobin disorders, which provided a detailed map of the organization and function of this enhancer (Canver et al., 2015). A similar approach was used to identify novel enhancers that regulate genes involved in BRAF inhibitor resistance in melanoma (Sanjana et al., 2016). Another study mutated p53 binding sites genome‐wide to understand enhancers that control oncogene‐induced senescence, a cell‐cycle arrest program induced by p53 (Korkmaz et al., 2016). A less biased approach is to make tiling deletions along a genomic region by delivering two sgRNAs to each cell (Figure 5d; Gasperini et al., 2017; Shen et al., 2017), which successfully identified novel enhancers of *POU5F1* using human embryonic stem cells (ESCs; Shen et al., 2017).

At present, a major limitation of CRISPR screens is that indels produced by NHEJ may not be sufficient to disrupt enhancer function, and even if function is disrupted by indels or larger deletions, this may fail to produce detectable phenotypes due to enhancer redundancy. Instead of phenotyping mutants based on discrete cellular phenotypes, some studies have utilized knock‐in GFP reporters as a quantitative and continuous readout of gene regulatory activity (Rajagopal et al., 2016; Shen et al., 2017). Furthermore, single cell RNA‐seq (scRNA‐seq) has recently been applied to pooled CRISPR screens, particularly those that cause multi‐locus perturbation, as it allows for comprehensive cell phenotyping (Dixit et al., 2016; Jaitin et al., 2016). Following large‐scale CRISPR/Cas9 screens, genome‐wide data from ChIP‐seq, ATAC‐seq, Hi‐C, and related methods are typically used to prioritize putative enhancers for targeted functional validation.

### 5.2 CRISPR activation and CRISPR interference to identify candidate enhancers

CRISPR screens that utilize variant forms of Cas9 also have the potential to be useful for the functional identification of putative enhancers. Catalytically dead Cas9 (dCas9) can be fused to an effector domain that either activates (CRISPRa) (Gilbert et al., 2014; Maeder et al., 2013; Perez‐Pinera et al., 2013; Figure 5e) or interferes with (CRISPRi) (Gilbert et al., 2013, 2014; Figure 5f) the expression of target genes. These Cas9 fusion proteins do not cut DNA, but instead, when paired with a specific sgRNA, recruit effector domains to specific genomic loci. CRISPRa strategies to activate gene expression commonly utilize dCas9 fused to the C‐terminal VP64 acidic transactivation domain derived from herpes simplex virus protein VP16 (Beerli, Dreier, & Barbas, 2000; Perez‐Pinera et al., 2013; Seipel, Georgiev, & Schaffner, 1992). A pooled screen successfully identified stimulus‐responsive enhancers when CRISPRa was recruited to the autoimmunity risk loci *CD69* and *IL2RA* (Simeonov et al., 2017).

A more popular approach for studying enhancer function has been CRISPRi, in which dCas9 is fused to a Krüppel‐associated box (KRAB) domain that represses gene expression (Gilbert et al., 2014). The KRAB domain works by recruiting repressive cofactors that methylate or deacetylate histones, which ultimately results in heterochromatin spreading and subsequent impacts on gene expression (Groner et al., 2010; Reynolds et al., 2012; Schultz, Ayyanathan, Negorev, Maul, & Rauscher, 2002; Sripathy, Stevens, & Schultz, 2006). A CRISPRi screen using tiled sgRNAs in the vicinity of *MYC* and *GATA1* loci revealed nine distal enhancers that control cellular proliferation (Fulco et al., 2016). Putative enhancers that correlated well with H3K27ac, Hi‐C data, and DNase hypersensitive sites were thoroughly validated by luciferase assays, qPCR for the predicted target gene, and loss of function experiments (Fulco et al., 2016). Other studies have used CRISPRi with multiple sgRNAs per cell to interrogate combinatorial enhancer activity, followed by scRNA‐seq to phenotype cells (Gasperini et al., 2019; Xie, Duan, Li, Zhou, & Hon, 2017). sgRNAs targeting 71 constituent enhancers from 15 super‐enhancers revealed some individual enhancer sequences that had strong effects on gene expression, while others only showed an effect when silenced in combination and thus appear to be redundant (Xie et al., 2017).

A recent CRISPRi screen delivered lentiviral sgRNA constructs at a high MOI to achieve an average of 28 random perturbations per cell (Gasperini et al., 2019). Rather than focusing on a particular gene or TF, this study silenced combinations of candidate enhancers genome‐wide and then used scRNA‐seq data to infer hundreds of putative enhancer‐gene regulatory relationships, a handful of which were validated by homozygous deletion of the enhancer and gene expression assays (Gasperini et al., 2019). As expected due to the redundancy of enhancer sequences, only 10% of candidate enhancers that were targeted by CRISPRi affected gene expression (Gasperini et al., 2019). Indeed, enhancer redundancy may significantly limit the ability of CRISPR perturbations to identify functional enhancers, demonstrating the challenge in identifying enhancers within the endogenous locus and highlighting the value of MPRAs as a complementary approach to study enhancer function.

### 5.3 Current limitations of high‐throughput CRISPR screens

Altogether, high‐throughput screens using CRISPR/Cas9 and its derivatives have allowed for the analysis of numerous *cis*‐regulatory elements in their native genomic context. Although several novel enhancers have been functionally identified, these methodologies are still in their infancy and there are several important considerations moving forward. CRISPR screens typically utilize data from ChIP‐seq, DNase‐seq, Hi‐C and related assays to prioritize hits. However, a CRISPR screen focused on ESC‐specific genes identified “unmarked” regulatory elements that lacked stereotypical epigenetic features of enhancers (Rajagopal et al., 2016). This finding highlights the value of pooled CRISPR screens as a complementary approach to identify candidate *cis*‐regulatory elements. Another study found that DNase I hypersensitive sites, Hi‐C interactions, and H3K27ac were able to predict enhancers from a CRISPRi screen, but ranked them in the wrong order (Fulco et al., 2016). For example, the enhancer with the most significant DNase‐seq peak had the smallest effect on gene expression. An additional consideration is that random indels produced by NHEJ may be insufficient to disrupt enhancer function. On the other hand, the heterochromatin spreading induced by dCas9‐KRAB can span across 1 to 2 kb (Gasperini et al., 2019), and thus lacks the resolution to discern which enhancer, or which parts of a putative enhancer sequence, are important for function. This could cause off‐target effects that are unrelated to sgRNA specificity. Even if a candidate enhancer sequence is successfully and specifically disrupted, perturbation of a single enhancer may not produce a detectable phenotype. Indeed, current CRISPR screens are likely unable to identify some functional enhancers because of enhancer redundancy. Finally, it remains unclear whether all enhancers are equally susceptible to perturbation by CRISPR or CRISPRi. Indeed, nucleosome positioning has been shown to play a key role in the ability of Cas9 to interact with DNA, and thus it may be difficult for Cas9 to act at enhancers located in nucleosome dense regions (Horlbeck et al., 2016).

Chromatin accessibility is also an important consideration when choosing a cell type for high‐throughput CRISPR screens, as results could be impacted by a non‐relevant chromatin context. A number of studies have employed the human immortalized myelogenous leukemia cell line K562 (Dixit et al., 2016; Fulco et al., 2016; Gasperini et al., 2019; Xie et al., 2017). These cells are advantageous because they can easily be grown in suspension and have been engineered to stably express Cas9 (Deans et al., 2016), but it is unclear whether findings in this cell line can be extrapolated to other cellular contexts. One strategy to mitigate this issue has been to do an initial CRISPR screen in K562 cells followed by a sub‐screen of the most significant hits in a more relevant cell type (Kramer et al., 2018). At present, large‐scale CRISPR screens may be particularly useful for identifying putative regulatory elements that lack the stereotypical epigenetic signatures associated with enhancers. These methodologies have just begun to be applied to the whole genome for the unbiased identification of candidate enhancer sequences (Gasperini et al., 2019), and will no doubt have a significant impact on our ability to understand enhancer function. Moving forward, it will be important to validate putative enhancer sequences found in high‐throughput CRISPR assays at scale. The sufficiency of these sequences to drive gene expression could be tested in MPRAs, while necessity could be assessed in scaled up CRISPR deletion assays. Thus, we predict that MPRAs and CRISPR screens will provide complementary insight into enhancer function and will together advance our ability to identify bona fide enhancer sequences.

### 5.4 CRISPR approaches in animal models

A primary advantage of CRISPR screens is the ability to identify and manipulate candidate enhancer sequences within the native chromatin context in a high‐throughput manner, but these studies currently lack any direct insight into development. Animal models remain a critical tool for validating putative enhancer sequences and understanding enhancer function within a developmental context, but there are significant challenges in scaling up these approaches. CRISPR screens have been performed in whole animals, in which exogenous, edited cancer cells are introduced into immunocompromised mice and scored for tumor growth and metastasis (S. Chen et al., 2015). However, at present the utility of this approach is likely limited to studies of hematopoiesis, blood disorders, and cancer.

Recently, a gene drive strategy was developed in mice that biases the inheritance of desired alleles and has the potential to greatly increase the efficiency with which transgenic mouse lines can be generated (Grunwald et al., 2019). This approach could be used to efficiently manipulate a handful of enhancer sequences at once, which would be particularly useful given the number of predicted redundant enhancers present in genomes. Furthermore, CRISPR strategies could be used alongside anti‐CRISPRs (Bubeck et al., 2018; Nakamura et al., 2019), phage‐derived proteins that abrogate CRISPR activity, to facilitate spatiotemporally restricted genomic perturbations during development. This approach would allow for finely tuned functional studies and could provide novel insight into enhancer function. High‐throughput CRISPR screens have rapidly provided a wealth of information regarding enhancer function and are a tantalizing method with which to study developmental enhancers, but there are still bottlenecks that must be overcome to gain direct insight into organismal development.

## 6 SINGLE‐CELL TRANSCRIPTOMIC APPROACHES TO CONSTRUCT ATLASES OF ANIMAL DEVELOPMENT

A major objective in developmental biology is to understand how changes in gene expression direct the progression of embryonic cell lineages from pluripotency to diverse cell fates. Because enhancers control patterns of gene expression during development, significant efforts have focused on locating enhancer sequences in the genome, elucidating the specific tissues in which they are active, and identifying the genes that they regulate. Although it remains a substantial challenge to directly link enhancer sequences to tissue‐specific gene expression at scale, single‐cell transcriptomics approaches promise to play an integral role in achieving this goal. Early scRNA‐seq was performed after FACS sorting (Meador et al., 2014) or microfluidics processing (e.g., the Fluidigm C1 platform) (Pollen et al., 2014) to isolate individual cells within separate compartments of a multiwell plate (F. Tang et al., 2009, 2010). While these methods offer some advantages, isolating individual cells can be expensive and time consuming (Kolodziejczyk, Kim, Svensson, Marioni, & Teichmann, 2015; Trapnell & Liu, 2016), limiting the number of single cells that can be analyzed in a single experiment. Recent advances in scRNA‐seq have greatly increased the number of cells that can economically be analyzed (J. Cao et al., 2017; Macosko et al., 2015), making it possible to assemble comprehensive, single‐cell transcriptional atlases of developing embryos. These developmental atlases provide a description of gene expression in each cell of a developing embryo, revealing the transcriptional blueprints for development.

**Figure 6:**
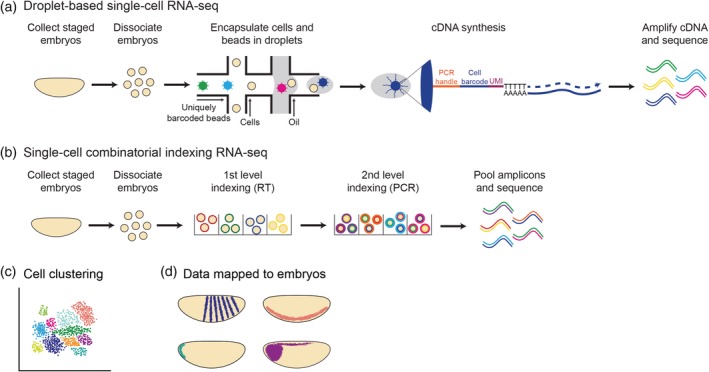
scRNA methodologies to build atlases of animal development. Single cell RNA‐sequencing (scRNA‐seq) methods used to generate atlases of animal development are illustrated using *Drosophila* as a model system. Staged embryos are collected and dissociated before being subjected to (a) droplet‐based scRNA‐seq or (b) single‐cell combinatorial indexing RNA‐seq (sciRNA‐seq). Droplet‐based scRNA‐seq methods utilize beads bearing primers that contain two unique barcodes, one that is cell‐specific and one that is transcript‐specific (Unique Molecular Identifier). The primers also contain a PCR handle for subsequent amplification. For sciRNA‐seq, cells are distributed to multiwell plates where they receive a well‐specific barcode during reverse transcription. Cells are then pooled and distributed to new multiwell plates where they receive a second well‐specific barcode during amplification. A third level of indexing can increase the number of cells processed in a single experiment (not shown). (c) Single‐cell transcriptomic data is represented as a t‐distributed stochastic neighbor embedding (t‐SNE) or uniform manifold approximation and projection (UMAP) plot, which cluster cells based on the similarity of their transcriptomes. (d) Cell type‐specific markers and in situ hybridization data can be used to map single‐cell transcriptomes back to a virtual embryo

To generate an atlas representing a developmental timepoint, embryos are dissociated into single cells, and then subjected to scRNA‐seq. scRNA‐seq is compatible with fixed cells, which can minimize the effects of dissociation and handling on cell state (J. Cao et al., 2017). In droplet‐based scRNA‐seq approaches, cells are introduced into a microfluidic system in which single cells are encapsulated in oil droplets containing uniquely barcoded primers on a bead (Macosko et al., 2015; Figure 6a). Primers contain two barcodes, one that is cell‐specific (i.e., represented on every primer for a given bead) and one that is transcript‐specific (i.e., unique to each primer on a given bead). mRNA is then reverse‐transcribed and amplified, after which barcoded DNA can be pooled and sequenced. An alternate method is single‐cell combinatorial indexing (sci)‐RNA‐seq (J. Cao et al., 2017), which utilizes split‐pool barcoding to uniquely label single cells (Figure 6b). Briefly, cells are distributed to multiwell plates, where a unique barcode is introduced to each well during reverse transcription (RT). Cells are then pooled and redistributed, and a second unique barcode is introduced to each well during PCR. Amplicons can then be pooled and subjected to massively parallel sequencing. Following scRNA‐seq by either method, single‐cell transcriptomic information is then deconvoluted using cell‐specific barcodes, and transcript abundance quantified using unique molecular identifiers. Computational approaches are then used for the unsupervised clustering of cells based on the similarity of their transcriptomes (Figure 6c; Kiselev, Andrews, & Hemberg, 2019). Cell clusters are typically represented as a t‐distributed stochastic neighbor embedding (t‐SNE) or uniform manifold approximation and projection plot (UMAP), the latter of which preserves data structure in such a way that inter‐cluster relationships can be elucidated (Becht et al., 2019). Because cells are dissociated, spatial information is lost, but can be reconstructed using known cell type‐specific markers and in situ hybridization data (Figure 6d).

### 6.1 Single‐cell transcriptomics can provide new insight into development

Single‐cell transcriptomic approaches were applied to the construction of a comprehensive developmental atlas for *C. elegans* (J. Cao et al., 2017). In L2 stage *C. elegans* embryos, many distinct neuronal cell types were identified, including some known to correspond to only one or two cells in the larval worm. The ability to detect extremely rare cell types highlights the detail with which this approach can map single‐cell transcriptomes from whole embryos. In *Drosophila*, this approach has been used to characterize stage 6 embryos, which represent the onset of gastrulation (Karaiskos et al., 2017). Single‐cell transcriptome data were correlated with high‐resolution in situ expression patterns for 84 marker genes, allowing single cells to be mapped to specific positions on a virtual embryo. This information was used to predict “virtual” in situ hybridization patterns for transcripts expressed in the stage 6 embryo, many of which were validated by in situ hybridization and found to be accurate across a wide range of expression patterns (Karaiskos et al., 2017). Interestingly, long noncoding RNAs (lncRNAs) were some of the most variable transcripts between clusters, and many exhibited patterned expression, suggesting that lncRNAs may have a role in early embryonic patterning and development (Karaiskos et al., 2017). Furthermore, the authors of this study identified patterned expression of Hippo signaling components, which are major regulators of organ size, cell cycle, and proliferation and had not previously been implicated in early embryonic development. Indeed, increased nuclear localization of Yorkie, a TF downstream of Hippo signaling, was found in cells that were undergoing mitosis, suggesting a new role of Hippo signaling in cell cycle regulation during early *Drosophila* development (Karaiskos et al., 2017). Thus, while scRNA‐seq of whole embryos is primarily a descriptive method, new biological insights are already being gleaned from these datasets.

Atlases of animal development have not yet been directly applied to the study of enhancers, although additional insight into developmental gene regulation could be achieved by combining scRNA‐seq with single‐cell ATAC‐seq to assay chromatin accessibility, which may reveal putative developmental enhancers. This principle was recently demonstrated in *Ciona* embryos by bulk RNA‐seq and ATAC‐seq performed on FACS sorted cardiac progenitor cells, which led to the identification of novel enhancers (Racioppi, Wiechecki, & Christiaen, 2019). Pairing single‐cell approaches within the context of a whole developing embryo will likely expand our ability to identify and functionally validate enhancer sequences.

### 6.2 Phenotyping mutants with single‐cell resolution

Beyond facilitating the construction of developmental atlases, scRNA‐seq allows for rapid, unbiased phenotyping of mutant embryos at single‐cell resolution. Several studies have directly compared the transcriptional landscapes of wildtype embryos and embryos bearing mutations in genes known to be important for development. Intriguingly, it appears thus far that single cells from mutant embryos adopt a subset of wildtype transcriptomic states, but do not exhibit markedly distinct transcriptomes compared to wildtype.

In maternal‐zygotic one‐eyed pinhead (MZ*oep*) mutant zebrafish, which are defective in nodal signaling, no mutant‐specific transcriptomic states were identified, although wildtype states were significantly reduced or absent (Farrell et al., 2018). Similarly, disruption of the *chordin* locus in zebrafish, which is required for patterning the early dorsal ventral axis (Sasai et al., 1994; Schulte‐Merker, Lee, McMahon, & Hammerschmidt, 1997), did not change the overall transcriptional landscape, but dramatically altered cell state abundances (Wagner et al., 2018). In mouse, scRNA‐seq was used to study an embryonic lethal mutation in *Tal1*, a gene that is critical for hematopoiesis (Pijuan‐Sala et al., 2019). Challenges in studying this lethal mutation were overcome by injecting tdTomato‐positive, homozygous *Tal1* mutant ESCs into wildtype blastocysts, and then dissociating and sorting cells from the resulting embryos prior to scRNA‐seq. Similar to findings in zebrafish, mutant cells did not form novel clusters. However, tdTomato‐positive, *Tal1*‐null cells from chimeric embryos were depleted of multiple blood cell clusters (Pijuan‐Sala et al., 2019).

Moving forward, comparing scRNA‐seq data from embryos bearing mutations within enhancers to stage‐matched wildtype transcriptomic atlases will be a valuable method for systematically assessing how enhancer sequences contribute to development on a global scale. This approach will provide unprecedented insight into the specific cell types that are affected by enhancer manipulations within the context of developing embryos.

### 6.3 Reconstruction of developmental trajectories

When cells from embryos representing different developmental timepoints are subjected to scRNA‐seq, trajectories of differentiation can be reconstructed over time. Single‐cell trajectories are built by a computational approach termed pseudotime ordering (Trapnell et al., 2014), which reflects developmental progress rather than absolute time. In zebrafish, many different embryonic stages have been evaluated by this method, in both wildtype and mutant animals (Farrell et al., 2018; Wagner et al., 2018). In wildtype zebrafish, trees constructed by pseudotime ordering substantially mirrored the developmental trajectories expected from classic embryological studies (Farrell et al., 2018). However, some branch points contained intermediate cells that expressed genes characteristic of many disparate cell fates (Farrell et al., 2018). One study complemented its reconstructed atlas of the zebrafish embryo with a transposon‐based lineage tracing approach to interrogate whether individual cell histories correlated with inferred developmental trajectories (Wagner et al., 2018). This revealed that some clonally distinct lineages can give rise to similar cell types, while in other cases, transcriptional changes can drive related cells toward distinct fates. This finding highlights the complexity of development and the need for complementary strategies to study developmental processes. While at present treelike hierarchies cannot fully represent the dynamics of differentiating cells in the zebrafish embryo, using single‐cell transcriptome analysis in combination with lineage tracing can help us to better understand developmental trajectories. If combined with single cell ATAC‐seq, this approach could contribute new insights into gene regulatory dynamics during development.

### 6.4 Methods to study developmental trajectories have broad implications

Moving forward, we predict that methods to reconstruct developmental trajectories will be broadly applicable to the study of biological processes that share similarities with development, including cancer progression and tissue regeneration. Indeed, regeneration trajectories have been described in the regenerative planarian *Schmidtea mediterranea*, an immortal organism that continuously regenerates its tissues and has remarkable regenerative capabilities (Plass et al., 2018). All cell types and trajectories identified in regenerating planarians were present in the intact planarian atlas, demonstrating that there are not regeneration‐specific trajectories. However, regenerating planarians showed a large increase in neoblast numbers, consistent with increased mitotic activity, and increased neural progenitor cells, reflecting active neurogenesis to replace missing head structures (Plass et al., 2018). This study also highlighted the potential of developmental atlases to rapidly accelerate our understanding of developmental processes in emergent model organisms.

The construction of developmental atlases may also be applicable to questions in evolutionary developmental biology. In a comparison of developmental trajectories from zebrafish and *Xenopus*, lineage topologies were found to be broadly conserved between species, while the observed abundances of different cell types were markedly different between species (Briggs et al., 2018). Furthermore, a recent study of developmental trajectories in *C. intestinalis* provided new insight into the origins of the vertebrate telencephalon (C. Cao et al., 2019). As more developmental atlases become available, more cross‐species comparisons can be made. These studies are undoubtedly just the first of a new wave of literature that will give significant insights into the transcriptomic dynamics of embryonic development, and will provide a foundation for future studies of enhancer function in development, regeneration, and disease.

### 6.5 Future directions in single‐cell studies of developing embryos

Several groups have demonstrated that these single‐cell methods can be performed in an efficient and cost‐effective manner, facilitating their application to the study of diverse developmental contexts. A current technical limitation is the inability to capture all of the different transcripts from a single cell, meaning that some transcripts will inevitably be undetected (J. Cao et al., 2017). However, developmental atlases of mid‐gestational mouse embryos revealed novel notochord markers (J. Cao et al., 2019), indicating that this lack of depth does not preclude new biological insights. Furthermore, rare cell types including germ cells have reproducibly been identified by scRNA‐seq of whole embryos (Briggs et al., 2018; J. Cao et al., 2019; Farrell et al., 2018; Pijuan‐Sala et al., 2019; Wagner et al., 2018), demonstrating that these methods can provide fine detail of single‐cell transcriptomes.

In future experiments, scRNA‐seq could be combined with single‐cell ATAC‐seq or Hi‐C to investigate the transcriptomic and chromatin landscapes of whole embryos. Although these combined approaches would not directly test enhancer function, they could identify putative developmental enhancers and provide insight into *cis*‐regulatory dynamics in developing embryos. Further functional insight could come from scRNA‐seq of embryos bearing mutations in enhancer sequences. Because spatial information is lost when embryos are dissociated, construction of developmental atlases currently requires human annotation based on known marker genes. While human annotation can be semi‐automated and has been useful for validating these methodologies, in the future this limitation could be overcome by methods that couple spatial and transcriptomic data. For example, multiplexed fluorescence in situ hybridization techniques allow for the detection of thousands of RNA species to be imaged in single cells in tissue sections (K. H. Chen, Boettiger, Moffitt, Wang, & Zhuang, 2015; Lubeck & Cai, 2012; Lubeck, Coskun, Zhiyentayev, Ahmad, & Cai, 2014; Moffitt et al., 2018). One of these methods, termed multiplexed error‐robust FISH (MERFISH), has been combined with scRNA‐seq to generate a comprehensive map of the hypothalamic preoptic region in mouse (Moffitt et al., 2018), an approach that could easily be extended to the study of whole embryos. Additionally, spatial transcriptomic methods have recently been developed that enable in situ barcoding and cDNA synthesis, such that high‐quality RNA‐seq data can be generated with retained spatial information (Ståhl et al., 2016). These methods will advance our ability to construct accurate and high‐resolution atlases of embryonic development. Looking ahead, we expect that advances in single‐cell transcriptomics approaches will have a substantial impact on our understanding of how enhancer sequences affect cell type‐specific gene expression in developing embryos.

## 7 CONCLUSION

Since the discovery of enhancers over 30 years ago, technological advancements have greatly improved our ability to identify and study enhancers. Advances in high‐throughput sequencing, oligonucleotide synthesis, and genome editing technology have enabled new methodologies that have been critical in elucidating how enhancer sequences relate to function. The development of genome‐wide assays has enabled the identification of millions of putative enhancer sequences that are associated with particular chromatin signatures. Genome‐wide assays have also led to an increased interest in understanding enhancer function because it is now clear that the majority of disease‐causing genetic variants reside within putative enhancer sequences.

Bottlenecks in our ability to functionally validate candidate enhancer sequences are starting to be overcome by MPRAs, although these are most valuable when done in a context‐specific way. Advantageously, MPRAs can be performed in whole embryos to give direct insight into tissue‐specific enhancer function during development. CRISPR screens are also useful because they allow enhancers to be identified and studied within the native genomic context, but such screens have not yet been done in developing embryos. The major challenge will be designing these in a way that is informative despite significant enhancer redundancy.

Similarly, a recent wave of developmental atlases has given unprecedented insight into the transcriptomes of whole developing organisms with single‐cell resolution, but these methods are primarily descriptive in their present state. Given the current limitations of these methods, animal models are still important for their direct relevance to development and have proven to be a valuable tool with which to correct misconceptions that have arisen from genome‐wide data. Moving forward, technological advances in high‐throughput CRISPR screens and single‐cell transcriptomic analyses of developing embryos will likely allow for the study of enhancer function in developmental contexts. Together with MPRAs, these approaches will allow us to gain a broad understanding of which enhancer sequences are functional and important for successful development.

## CONFLICT OF INTEREST

The authors have declared no conflicts of interest for this article.

## AUTHOR CONTRIBUTIONS

**Genevieve E. Ryan**: Conceptualization; writing‐original draft; writing‐review and editing. **Emma K. Farley**: Conceptualization; writing‐original draft; writing‐review and editing.

## RELATED WIREs ARTICLE

https://dx.doi.org/10.1002/wsbm.1390
